# Associations Between Nutrient Intake and Cognitive Performance Across Multiple Domains in Healthy School-Aged Children

**DOI:** 10.3390/nu18193186

**Published:** 2026-09-26

**Authors:** Khitam Muhsen, Ashraf Akawi, Rebecca Goldsmith, Dina Levy, Nuha Zeidan, Asher Ornoy, Eias Kassem, Wasef Na’amnih

**Affiliations:** 1 Department of Epidemiology and Preventive Medicine, School of Public Health, Gray Faculty of Medical and Health Sciences, Tel Aviv University, Tel Aviv 6139001, Israelwasefnammnih@tauex.tau.ac.il (W.N.); 2 Child Development Center, Hillel Yaffe Medical Center, Hadera 3810101, Israel; 3 Nutrition Division, Ministry of Health, Jerusalem 9101002, Israel; 4 Diet and Nutrition Unit, Clalit Health Service, Arara 30026, Israel; 5 Adelson School of Medicine, Ariel University, Ariel 4077625, Israel; asher.ornoy@mail.huji.ac.il; 6 Department of Pediatrics, Hillel Yaffe Medical Center, Hadera 3810101, Israel

**Keywords:** dietary intake, cognitive function, dietary fiber, fatty acids, school-aged children

## Abstract

**Background and objective:** Evidence regarding dietary intake of specific macronutrients, dietary fiber, and fatty acids in relation to cognitive performance during school age remains limited. We examined these associations among healthy Arab children in Israel. **Methods:** This cross-sectional analysis included 146 children aged 10–12 years from a prospective cohort. Dietary intake was assessed using a single 24 h dietary recall. Cognitive performance was assessed using the Arabic version of the Wechsler Intelligence Scale for Children, Fourth Edition. Full-scale intelligence quotient (FSIQ) was the primary outcome, whereas verbal comprehension (VCI), perceptual reasoning (PRI), working memory (WMI), and processing speed (PSI) indices were exploratory secondary outcomes. Multivariable linear regression models adjusted for age, sex, village, socioeconomic status, and total energy intake. **Results:** No statistically significant associations were found between the examined dietary exposures and FSIQ scores. Among 48 exploratory comparisons involving 12 dietary exposures and four specific cognitive domains, higher dietary fiber intake was associated with higher WMI (B = 0.430, 95% CI 0.067–0.794), and this association remained consistent across sensitivity analyses. Higher polyunsaturated fatty acid intake was associated with higher PRI (B = 0.312, 95% CI 0.021 to 0.604), and higher linolenic acid intake with higher PSI (B = 3.325, 95% CI 0.081 to 6.569). However, both findings were sensitive to alternative exposure specifications. In a sensitivity analysis, any calculated trans-fat intake versus zero calculated intake was associated with lower PRI (B = −6.290, 95% CI −10.766 to −1.814). **Conclusions:** We found no evidence of associations between the examined nutrient intakes and FSIQ, the primary outcome for overall intellectual functioning. Exploratory analyses identified domain-specific associations, particularly a consistent positive association between dietary fiber intake and working memory. These findings provide new evidence on nutrient intake and cognitive performance among healthy school-aged children from an underrepresented population. Given the study limitations, these associations should be interpreted cautiously and confirmed in larger prospective studies using repeated dietary assessments.

## 1. Introduction

Childhood is characterized by ongoing structural and functional brain maturation that occurs alongside the development of increasingly complex cognitive abilities [1,2]. Adequate dietary intake is essential for brain development, providing energy and critical nutrients that support multiple neurodevelopmental processes [3,4]. Although associations between nutrition and neurodevelopment have been studied extensively during fetal life and early childhood [3], brain maturation continues into later childhood [1], raising the possibility that dietary intake during school age may be a modifiable factor influencing cognitive performance.

Observational studies in healthy children have examined the associations between diet and cognitive development using two broad approaches: overall dietary patterns [5,6,7] and specific dietary components [8,9]. Healthier dietary patterns have been associated with better cognitive performance, whereas patterns characterized by processed, energy-dense, or high-sugar foods have been linked to poorer cognitive outcomes [5,6,7,10]. However, dietary patterns do not identify the specific nutrients that may underlie these associations. Relatively few studies in healthy school-aged children have examined quantitative intake of specific macronutrients, dietary fiber, and fatty acids in relation to intelligence or standardized cognitive performance [8,9].

Available evidence suggests that associations with specific dietary components may differ across cognitive domains [9,11,12,13]. A large US study of 3666 children aged 6–16 years showed associations between selected dietary fats and specific cognitive measures, but not with overall intelligence and psychosocial functioning [9]. Specifically, higher polyunsaturated fatty acid (PUFA) intake was associated with better performance on the digit span test, whereas higher cholesterol intake was associated with poorer performance [9]. Others reported associations between dietary fiber and cognitive control among healthy children [12]. Dietary intake of fatty acids has been differentially associated with memory performance: higher omega-3 fatty acid intake, a PUFA subclass, was associated with better relational memory, while higher saturated fatty acid intake was associated with poorer memory performance [11].

Despite this evidence, important knowledge gaps remain in understanding the role of diet in children’s cognitive function. Previous studies vary markedly in age and developmental stage, timing of dietary exposure, dietary assessment methods, and cognitive outcomes [5,6,7,8,9]. Several prospective studies have examined associations between dietary patterns in early childhood and cognitive development in later childhood [6,14,15]. However, evidence regarding concurrent nutrient intake during the school-age years remains limited. Dietary intake has also commonly been assessed using food frequency questionnaires [5,6,7], which are useful for characterizing habitual dietary patterns but provide less detailed quantitative information on nutrient intake than dietary recalls. Furthermore, cognitive assessment methods varied considerably across studies, ranging from standardized intelligence quotient (IQ) measures to tests of specific cognitive domains [8,9,11,12,13], thereby limiting direct comparisons.

Evidence from Middle Eastern populations is particularly scarce, including among minority groups with distinct sociocultural and dietary characteristics. In Israel, dietary intake among Arab children may differ from that reported in populations represented in previous studies and includes elements consistent with the Mediterranean diet [16]. We have demonstrated that early-life exposures and socioeconomic status (SES) were associated with cognitive development in Israeli Arab children [17]; however, the role of nutrient intake in cognitive performance in this population has not been examined. Therefore, this study aimed to examine the associations of dietary intake of macronutrients, dietary fiber, and fatty acids with cognitive performance among healthy Arab children aged 10–12 years in Israel. Full Scale IQ (FSIQ) was specified as the primary cognitive outcome, with specific cognitive domains examined as secondary outcomes. Based on previous evidence, we hypothesized that higher dietary fiber and PUFA intake would be associated with better cognitive performance, whereas higher saturated fat and cholesterol intake would be associated with poorer cognitive performance. Associations involving the remaining dietary exposures were considered exploratory.

## 2. Materials and Methods

### 2.1. Study Population and Design

The study population and design have been described previously [17,18,19,20,21]. This cross-sectional analysis included 146 Arab children aged 10–12 years who participated in a preadolescent follow-up conducted between 2017 and 2019 and had available data on dietary and cognitive assessments. Children with documented developmental delays or those enrolled in special-needs education programs because of developmental difficulties were excluded from the present study. The children were drawn from a prospective cohort established in 2007 among infants aged 1–9 weeks in two Arab villages in the Hadera subdistrict of Israel. The original cohort included singleton infants with a birth weight > 2 kg and gestational age > 34 weeks who had no prenatal or perinatal complications or diagnoses that might affect development [17,21]. The two villages differed in their socioeconomic profiles: village A had higher socioeconomic indicators than village B, although both had lower socioeconomic status than the overall Israeli population [22].

Of the 233 children in the original cohort (all aged 10–12 years), 53 did not enter the follow-up: two did not meet the inclusion criteria due to developmental delays, 18 declined to participate, 24 could not be located, and nine withdrew. Among the 180 enrolled children, 174 completed the dietary assessment. Of these, 28 did not complete the cognitive assessment despite repeated invitations, leaving 146 children with data from both dietary and cognitive assessments for the present study (Figure 1).

### 2.2. Data Collection

At the preadolescent follow-up, following written informed consent, trained interviewers conducted face-to-face interviews in Arabic with parents to obtain updated information on sociodemographic characteristics and the child’s health status. During subsequent visits, dietary intake was assessed using a structured 24 h dietary recall as described below, and anthropometric measurements were obtained by trained study personnel. Cognitive performance was assessed during a designated visit.

### 2.3. Cognitive Assessment

Cognitive performance was assessed by two trained Arabic-speaking psychologists, blinded to participants’ background characteristics, using the validated Arabic version of the Wechsler Intelligence Scale for Children—Fourth Edition (WISC-IV) [17,23,24,25]. The WISC-IV yields a FSIQ, representing overall intellectual functioning, and four index scores assessing specific cognitive domains: verbal comprehension index (VCI), perceptual reasoning index (PRI), working memory index (WMI), and processing speed index (PSI) [23]. FSIQ was specified as the primary cognitive outcome, while VCI, PRI, WMI, and PSI were examined as secondary outcomes to explore whether associations with dietary exposures varied across cognitive domains. All cognitive scores were analyzed as continuous variables.

### 2.4. Dietary Intake

Dietary intake during preadolescence was assessed by trained Arabic-speaking interviewers using a single interviewer-administered 24 h dietary recall conducted jointly with the child and the mother, as described previously [18,20]. The recalled day was a regular weekday during the school period. The recall was administered using a three-pass interview procedure to obtain information about all foods and beverages the child consumed during the preceding 24 h. The three passes were components of the same dietary recall and did not represent repeated assessments on separate days. During the first pass (“quick list”), the child and mother listed everything the child had eaten or drunk. During the second pass (“detailed description”), the interviewer obtained detailed information about each reported item, including the ingredients, preparation methods, and recipes of mixed dishes. During the third pass (“review”), the interviewer reviewed the complete list, probed for potentially omitted foods and eating occasions, and clarified portion sizes. The Israeli Ministry of Health Food and Food Quantities Guide, which includes photographs of commonly consumed Israeli foods, was used to assist with food identification and portion-size estimation. Household measurement aids, including cups, teaspoons, and tablespoons, were also used. The reported foods, beverages, mixed dishes, ingredients, and consumed quantities were entered into the Israeli TZAMERET nutrient-analysis software (version 4) by a study researcher (R.G.). The software generated estimates of total daily energy intake and daily intakes of macronutrients, dietary fiber, cholesterol, and fatty acids based on the type and quantity of each reported food or ingredient. A trans-fat intake value of zero indicated that none of the foods reported during the recalled day contained trans-fat according to the TZAMERET nutrient database; no cutoff was used to classify low intake values as zero. The dietary exposures examined in this study were protein, total fat, carbohydrate, dietary fiber, total sugars, saturated fat, cholesterol, monounsaturated fatty acids (MUFA), polyunsaturated fatty acids (PUFA), trans fatty acids, linoleic acid, and linolenic acid. Nutrient intakes were analyzed as continuous variables in their original units (g/day, except cholesterol, which was expressed as mg/day).

### 2.5. Covariates

Sociodemographic characteristics were considered potential confounders given their associations with both dietary intake and cognitive development [26,27]. Sociodemographic variables included the child’s age at the preadolescent assessment, sex, village of residence, and SES score. SES was represented by a composite score derived from factor analysis [20], incorporating residential SES rank, reported paternal years of education, household crowding, and household income. The resulting SES score was analyzed as a continuous variable, with higher scores indicating higher SES.

### 2.6. Other Measurements

#### 2.6.1. Anthropometric Assessment

Body weight was measured to the nearest 0.1 kg using a calibrated Tanita digital scale, with children wearing light clothing and no shoes. Standing height was measured to the nearest 0.1 cm using a stadiometer. Body mass index (BMI) was calculated as weight (kg)/height squared (m^2^). Height-for-age z-scores (HAZ) and BMI-for-age z-scores (BMIZ) were calculated using the 2007 World Health Organization growth reference for 5–19-year-old children and adolescents [28,29].

#### 2.6.2. Blood Biomarkers

Non-fasting peripheral blood samples were collected during the preadolescent follow-up. Hemoglobin, serum ferritin, vitamin B12, and folate concentrations were measured at the Hillel Yaffe Medical Center laboratory using standardized laboratory procedures [19].

### 2.7. Statistical Analysis

Continuous variables were described using means and standard deviations (SD) for approximately normally distributed variables and medians and interquartile ranges (IQR) for skewed variables. Categorical variables were summarized as frequencies and percentages. Characteristics of children who participated in the preadolescent follow-up were compared with those of eligible cohort members who did not participate to assess potential differences related to follow-up participation.

Associations between nutrient intake and cognitive performance were examined using multivariable linear regression. FSIQ was prespecified as the primary cognitive outcome, whereas VCI, PRI, WMI, and PSI were considered secondary, exploratory outcomes. Dietary exposures were modeled separately as continuous variables. For each nutrient–cognition association, two models were fitted. Model 1 adjusted for age at the preadolescent assessment, sex, village of residence, and SES score. Model 2, considered the primary fully adjusted model, additionally adjusted for total daily energy intake (kcal/day). The village of residence was retained in addition to the SES score because the two study communities differed in dietary intake and cognitive performance, and these differences may reflect contextual factors not fully captured by the individual-level SES measure. Results are presented as unstandardized regression coefficients (B) with 95% confidence intervals (CI) and two-sided *p*-values.

The Benjamini–Hochberg procedure [30] was used to account for multiple comparisons for the primary outcome and control the false discovery rate (FDR) across the prespecified nutrient-FSIQ associations from Model 2. Nominal two-sided *p*-values < 0.05 were considered statistically significant before FDR correction, and FDR-adjusted results were used when interpreting the primary FSIQ analyses. Analyses of VCI, PRI, WMI, and PSI were considered exploratory and were interpreted with particular attention to the magnitude and precision of effect estimates, consistency across model specifications, and the number of comparisons performed.

Model assumptions and influential observations were evaluated using residual diagnostics, including standardized and studentized deleted residuals, Cook’s distance, leverage values, and collinearity statistics. Variance inflation factor (VIF) and tolerance values were examined to assess multicollinearity.

Sensitivity analyses were conducted for the three nutrient–cognitive–domain associations that were nominally statistically significant (*p* < 0.05) in the fully adjusted Model 2: dietary fiber with WMI, PUFA with PRI, and linolenic acid with PSI. The alternative exposure specifications were selected according to the distributions and characteristics of the respective nutrients. Because dietary fiber, PUFA, and linolenic acid intakes were right-skewed, the corresponding models were repeated after natural-log transformations to assess whether the findings were sensitive to the exposure scale and to high intake values. PUFA and linolenic acid were also expressed as percentages of total energy intake to assess the robustness of the findings to energy-standardized definitions of fatty acid intake. When regression diagnostics identified influential observations, the corresponding models were repeated after excluding those observations. Because trans-fat intake was highly zero-inflated, with more than half of participants having a calculated intake of zero, it was additionally modeled as a binary variable comparing any intake to zero calculated intake. These analyses were considered exploratory sensitivity analyses rather than primary hypothesis tests.

The primary regression analyses were conducted using a complete-case approach, in which participants with missing values (*n* = 4) in the independent variables were excluded. Data were analyzed using IBM SPSS Statistics, version 31 (IBM, Armonk, New York, NY, USA) software.

### 2.8. Power Analysis

An a priori power assessment was conducted based on the anticipated sample of 146 participants and the expected correlation between dietary fat intake and IQ scores, treated as continuous variables. Assuming a two-sided α of 0.05, the study had 67.8% power to detect a small correlation (r = 0.20) and 95.9% power to detect a moderate correlation (r = 0.30). Thus, the available sample had limited power to detect small associations but adequate power to detect moderate associations.

### 2.9. Declaration of Using Generative AI and AI-Assisted Technologies

During the preparation of this manuscript, the authors used ChatGPT (version 5 education) and Grammarly (V1.2.298.1959 EDU) for language editing. After using these tools/services, the authors reviewed and edited the content as needed and take full responsibility for the content of the published article.

## 3. Results

Overall, 146 participants with a mean age of 11.3 years were included in this study (Figure 1). Participants who completed the preadolescent follow-up were comparable to those (*n* = 87) who did not in baseline characteristics. The only statistically significant difference was village of residence, with a higher proportion of children from the lower-SES village among those who completed the follow-up (Supplementary Table S1). Among participants with available anthropometric data (*n* = 141), 14.2% were classified as overweight and 44.0% as having obesity (Table 1).

Median daily energy intake was 1747 kcal, with carbohydrates, fat, and protein contributing 52.9%, 31.0%, and 15.1% of total energy, respectively (Table 2).

### 3.1. Associations Between Dietary Intake and Cognitive Performance

#### 3.1.1. Primary Cognitive Outcome: Full-Scale IQ

No significant associations were found between the examined dietary exposures and FSIQ in either Model 1 or Model 2 (Table 3). These findings remained unchanged after correction for multiple comparisons (Supplementary Table S2).

#### 3.1.2. Secondary Cognitive Outcomes

In the exploratory analyses of the four secondary cognitive outcomes, none of the dietary exposures was associated with VCI. In the fully adjusted models, higher PUFA intake was associated with higher PRI, higher dietary fiber intake with higher WMI, and higher linolenic acid intake with higher PSI. No other statistically significant associations were observed in the fully adjusted models (Table 4, Table 5 and Table 6 and Supplementary Table S3).

### 3.2. Sensitivity Analyses

Sensitivity analyses were conducted for the associations observed with the secondary cognitive outcomes. The positive association between dietary fiber intake and WMI was robust to alternative model specifications. The association remained statistically significant when dietary fiber was log-transformed (B = 7.412, 95% CI 1.455 to 13.368) and after exclusion of influential observations identified using Cook’s distance (B = 0.362, 95% CI 0.005 to 0.720). For linolenic acid and PSI, exclusion of the identified PSI outlier strengthened the positive association (B = 3.783, 95% CI 0.656 to 6.910). However, the association was attenuated when linolenic acid intake was log-transformed (B = 4.416, 95% CI −0.678 to 9.510) or expressed as a percentage of total energy intake (B = 5.453, 95% CI −1.279 to 12.184). Similarly, the positive association between PUFA intake and PRI was sensitive to the specification of exposure. When PUFA intake was expressed as a percentage of total energy intake, the direction of association remained positive but was attenuated (B = 0.612, 95% CI −0.092 to 1.316), whereas no significant association was observed when PUFA intake was log-transformed (B = 1.384, 95% CI −1.681 to 4.449) (Supplementary Table S4). Regression diagnostics showed no evidence that the primary PUFA–PRI association was driven by influential observations.

Given the highly zero-inflated distribution of trans-fat intake, with 57.5% of participants having a calculated intake of 0 g/day, trans-fat intake was additionally examined as a binary exposure comparing any calculated intake (>0 g/day) with zero calculated intake. Any trans-fat intake was associated with lower PRI scores in both Model 1 (B = −6.292, 95% CI −10.703 to −1.882) and Model 2 (B = −6.290, 95% CI −10.766 to −1.814) (Supplementary Table S4).

## 4. Discussion

In this cross-sectional study of Arab children aged 10–12 years, we examined the associations of dietary intake of macronutrients, dietary fiber, and fatty acids with FSIQ and specific cognitive domains. The main findings were that none of the examined dietary components was significantly associated with the prespecified primary cognitive outcome, FSIQ. However, among 48 exploratory analyses involving four domain-specific cognitive outcomes, three significant associations were observed. Higher dietary fiber intake was associated with higher WMI, higher PUFA intake with higher PRI, and higher linolenic acid intake with higher PSI. The dietary fiber–WMI association was the most consistent across model specifications and sensitivity analyses, whereas the PUFA–PRI and linolenic acid–PSI associations were sensitive to alternative exposure specifications. A separate exploratory analysis also suggested an inverse association between any calculated trans-fat intake and PRI. Given the multiple comparisons performed, these domain-specific findings may reflect chance and should be considered hypothesis-generating.

The positive association between dietary fiber intake and working memory was the most consistent domain-specific finding. Higher fiber intake was associated with higher WMI scores both before and after adjustment for total energy intake. Moreover, the association persisted after log transformation and exclusion of influential observations. This finding is broadly consistent with that reported by Khan et al. [12] who showed that higher dietary fiber intake among 7–9-year-old children was associated with better performance on a cognitive control task [12]. Although cognitive control and working memory are distinct cognitive functions, both contribute to executive functioning [31]. However, Khan et al. [12] did not specifically assess working memory using the WISC-IV; thus, direct comparability is limited. The present finding, therefore, provides preliminary support for further investigation of dietary fiber in relation to specific aspects of cognitive performance in children, but does not establish a robust or causal association.

The associations between fatty-acid intake and specific cognitive domains were less consistent. Previous observational studies have also linked PUFA intake with selected aspects of cognitive performance in children. Zhang et al. [9], in a large study of US children aged 6–16 years, reported an inverse association between PUFA intake (expressed as percentage of energy intake) and poor performance on the digit-span performance test. In a small-scale study of 52 children aged 7–9 years, Baym et al. [11] reported a positive association between dietary intake of omega-3 fatty acids, a subclass of PUFA, and hippocampal-dependent relational memory. However, these outcomes differ from PRI and PSI and therefore provide only indirect context for the present findings. Moreover, the PUFA–PRI and linolenic acid–PSI associations were attenuated when the exposures were log-transformed or expressed as percentages of total energy intake. The inverse association between calculated trans-fat intake and PRI was observed only when trans-fat intake was modeled as a binary exposure, given its highly zero-inflated distribution. Evidence concerning trans-fat intake and cognitive performance in healthy school-aged children remains limited. Collectively, the limited robustness of these fatty-acid findings precludes firm conclusions.

The lack of significant associations between the examined dietary exposures and FSIQ is the primary finding of the current study. At the same time, the exploratory domain-specific findings suggest that potential dietary associations may not be uniform across all aspects of cognitive performance. Previous studies have similarly reported associations between dietary components and specific cognitive functions, including memory, cognitive control, cognitive flexibility, and digit-span performance [9,11,12,13]. In the present study, the association between dietary fiber intake and working memory was the most consistent across model specifications and sensitivity analyses, whereas the fatty-acid findings were less robust. Given the number of comparisons performed and the sensitivity of some findings to alternative exposure specifications, the domain-specific results should be considered hypothesis-generating. Nevertheless, they support the value of assessing both global and domain-specific cognitive outcomes and provide specific hypotheses for investigation in larger prospective studies.

The descriptive characteristics of the study sample also require consideration. The standard deviations of the cognitive scores ranged from 13.6 to 17.1 and were broadly comparable to the expected WISC standard-score SD of 15, indicating heterogeneity in cognitive performance. The absolute cognitive scores should be interpreted within the socioeconomic and sociocultural context of the two relatively low-SES communities and likely do not reflect cognitive performance in the broader Arab population in Israel. The prevalence of overweight and obesity was high, at 14.2% and 44.0%, respectively. This burden of obesity was previously documented in the same cohort and examined in relation to early-life feeding practices and school-age dietary intake [18]. It represents an important aspect of the nutritional and socioeconomic context of the participating communities and should be considered when interpreting the present findings and their generalizability.

Our study has several strengths. Cognitive performance was assessed using the Arabic version of the WISC-IV by trained Arabic-speaking psychologists who were blinded to participants’ background characteristics, providing standardized measures of cognitive performance. Dietary intake was assessed using a single interviewer-administered 24 h recall conducted jointly with the children and their mothers. A structured three-pass interview procedure and standardized aids for estimating portion sizes were used to obtain detailed quantitative information on nutrient intake during the recalled day. The study also contributes evidence from an underrepresented population and included children from two communities with different socioeconomic profiles. The relatively narrow age range reduced heterogeneity related to developmental stage, and the analyses accounted for important sociodemographic factors, including age, sex, SES, and village of residence. Moreover, distinguishing a prespecified primary cognitive outcome from exploratory secondary outcomes, applying a correction for multiple comparisons to the primary FSIQ analyses, and conducting targeted sensitivity analyses enabled explicit evaluation of the robustness of the observed associations.

Our study also has limitations. Dietary intake was assessed using a single 24 h recall. Although this method provides detailed quantitative information on nutrient intake during the recalled day, it cannot characterize an individual child’s usual or long-term intake or account for variation between weekdays and weekends or across school and non-school periods. Day-to-day variation may therefore have resulted in exposure misclassification and attenuation of the observed associations. The modest sample size is another limitation. Although the study had adequate power to detect moderate associations, statistical power was limited for small associations, which may therefore have remained undetected. The participants were also generally healthy children aged 10–12 years. This age follows the period of most rapid early-life brain growth, and the generally healthy status of the participants may have reduced variation in nutritional and cognitive status and the likelihood of observing strong associations. Nevertheless, cognitive maturation continues during the school-age years [1,2]. Furthermore, residual confounding and reverse causation should also be considered. The models adjusted for age, sex, village, SES, and total energy intake; however, unmeasured or imperfectly measured factors, including the home learning environment, parental cognitive characteristics, broader dietary patterns, and other health-related behaviors, may have influenced both dietary intake and cognitive performance. The cross-sectional design precludes establishing temporality or causal relationships between dietary intake and cognitive performance. Cognitive or behavioral characteristics of the children, as well as associated family practices, may influence food choices, rather than the observed associations necessarily representing an effect of nutrient intake on cognitive performance.

Multiple comparisons represent an additional limitation. FSIQ was prespecified as the primary outcome, and the corresponding Model 2 nutrient analyses were corrected for multiple comparisons. However, analyses of VCI, PRI, WMI, and PSI were exploratory and were not multiplicity-adjusted. The domain-specific findings should be interpreted cautiously. Only three of the 48 exploratory nutrient–cognitive–domain comparisons were nominally statistically significant, and no multiplicity adjustment was applied to these secondary analyses. The small number of observed associations may reflect chance, particularly given that some findings were sensitive to alternative exposure specifications. Although the dietary fiber–WMI association was more consistent across sensitivity analyses, it also remains exploratory and requires independent confirmation. Finally, participants were recruited from two Arab communities in Israel, and the findings may not be generalizable to children from other socioeconomic, cultural, or dietary settings. The absolute cognitive scores should be interpreted within the socioeconomic and sociocultural context of the two relatively low-SES communities and likely do not represent cognitive performance in the broader Arab population in Israel. Although participants in the preadolescent follow-up were generally similar to eligible nonparticipants on the baseline characteristics examined, participation differed by village of residence.

## 5. Conclusions

In conclusion, no statistically significant associations were found between the examined dietary intake of the macronutrients, dietary fiber, and fatty acids and FSIQ scores, the predetermined primary measure of global intellectual functioning. Exploratory analyses suggested possible domain-specific associations, with the positive association between dietary fiber intake and working memory being the most consistent across model specifications and sensitivity analyses; associations involving fatty acid intake were less robust. In view of the limitations of the study, these exploratory findings should be interpreted with caution and considered hypothesis-generating. Nevertheless, this study provides initial evidence regarding nutrient intake and cognitive performance in an underrepresented population and identifies specific hypotheses for confirmation in larger prospective studies that use repeated dietary assessments.

## Figures and Tables

**Figure 1:**
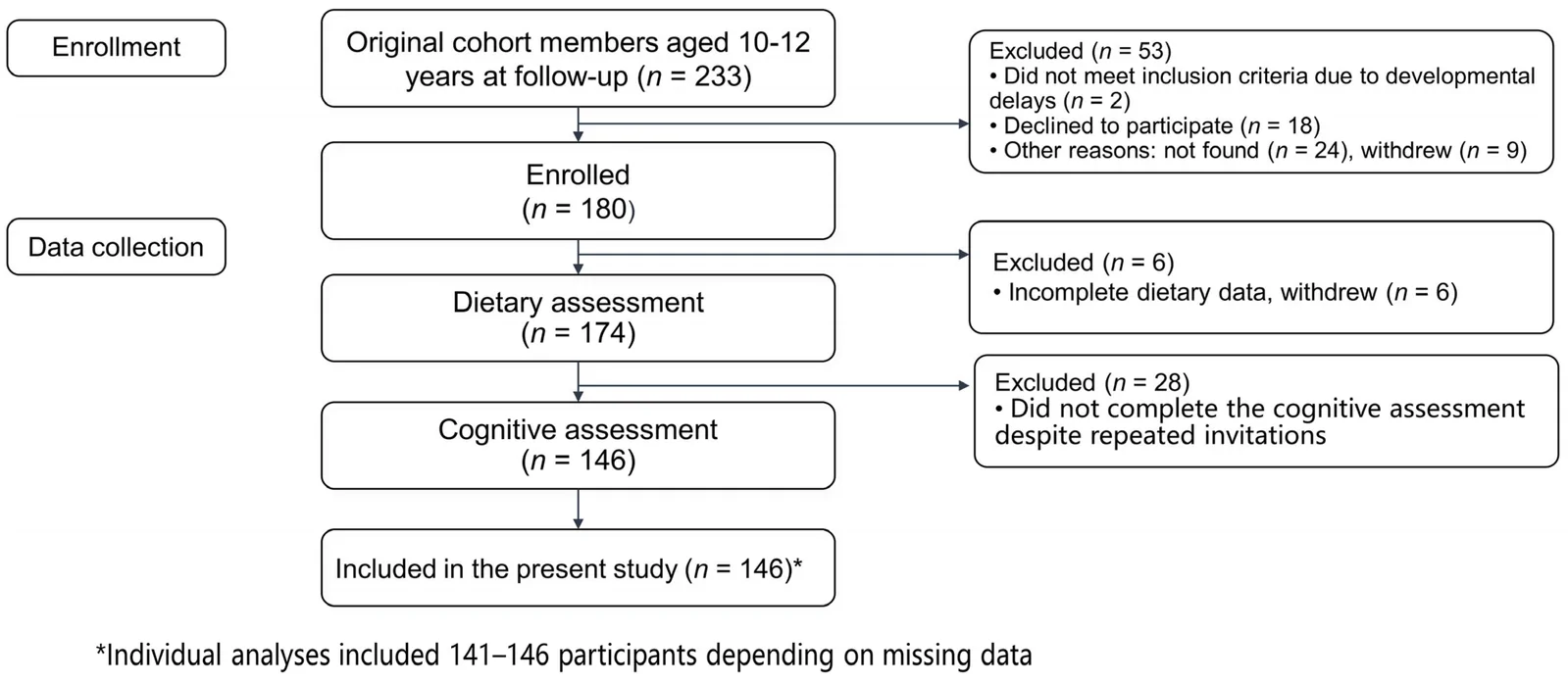
Flow chart of participant enrolment and inclusion in the present analysis.

**Table 1:** Characteristics of children participating in the preadolescent follow-up *.

Characteristic	Value
Sociodemographic characteristics	
Age, years, mean (SD)	11.3 (0.5)
Sex, *n* (%)	
Male	85/146 (58.2)
Female	61/146 (41.8)
Village of residence, *n* (%)	
Higher-SES village (A)	54/146 (37.0)
Lower-SES village (B)	92/146 (63.0)
Composite SES z-score	0.0 (1.1)
Anthropometric characteristics	
Weight, kg, mean (SD)	54.7 (18.3)
Height, cm, mean (SD)	152.1 (8.5)
Height-for-age z-score, mean (SD)	0.6 (1.2)
BMI-for-age z-score, mean (SD)	1.5 (1.5)
BMI-for-age category, *n* (%)	
Normal weight	59/141 (41.8)
Overweight	20/141 (14.2)
Obesity	62/141 (44.0)
Cognitive performance, mean (SD)	
Full Scale IQ (FSIQ)	84.1 (15.1)
Verbal Comprehension Index (VCI)	87.0 (13.6)
Perceptual Reasoning Index (PRI)	88.6 (17.1)
Working Memory Index (WMI)	85.3 (15.4)
Processing Speed Index (PSI)	85.2 (16.9)
Levels of blood biomarkers	
Hemoglobin, g/dL, mean (SD)	12.5 (0.9)
Serum iron, µg/dL, median (IQR)	60.5 (34.3)
Ferritin, ng/mL, median (IQR)	45.2 (33.4)
Vitamin B12, pg/mL, median (IQR)	501.3 (246.6)
Folate, ng/mL, median (IQR)	7.3 (3.9)

* information on age was missing for one participant, on socioeconomic status for three participants, on weight for five participants, on height for four participants, and on blood biomarkers for six participants. BMI: body mass index; cm: centimeter; IQR: interquartile range; kg: kilogram; SD: standard deviation; SES: socioeconomic status.

**Table 2:** Dietary intake at the preadolescent follow-up (*n* = 146).

Nutrient	Median (IQR)
Energy and macronutrients	
Energy (kcal/day)	1747.0 (804.5)
Protein (g/day)	63.8 (43.7)
Total fat (g/day)	56.5 (30.8)
Carbohydrates (g/day)	218.0 (98.6)
Dietary fiber (g/day)	16.5 (10.1)
Total sugars (g/day)	67.8 (55.3)
Fatty acids and cholesterol	
Cholesterol (mg/day)	164.4 (219.0)
Saturated fat (g/day)	17.9 (11.4)
Monounsaturated fat (g/day)	20.8 (13.5)
Polyunsaturated fat (g/day)	10.8 (13.0)
Trans fatty acids (g/day) *	0.0 (0.1)
Linoleic acid (g/day)	11.1 (8.7)
Linolenic acid (g/day)	1.5 (1.2)
Energy composition (% of total energy)	
Energy from fat (%)	31.0 (7.9)
Energy from carbohydrates (%)	52.9 (9.9)
Energy from protein (%)	15.1 (6.4)
Energy from saturated fat (%)	9.7 (4.9)
Energy from monounsaturated fat (%)	11.4 (4.2)
Energy from polyunsaturated fat (%)	5.9 (5.7)

* for trans-fat, 0 g/day indicates that none of the foods reported during the recalled day contained trans-fat according to the TZAMERET nutrient database.

**Table 3:** Adjusted associations of macronutrients, fiber, and fatty acids with the primary outcome, FSIQ.

	Model 1			Model 2		
Nutrient	B	95% CI	*p*-Value	B	95% CI	*p*-Value
Protein	−0.008	−0.073 to 0.057	0.815	−0.017	−0.110 to 0.077	0.727
Total fat	−0.006	−0.078 to 0.065	0.861	−0.028	−0.174 to 0.118	0.703
Carbohydrate	0.002	−0.025 to 0.029	0.872	0.006	−0.042 to 0.055	0.791
Dietary fiber	0.162	−0.114 to 0.438	0.248	0.215	−0.105 to 0.535	0.186
Total sugars	−0.021	−0.075 to 0.032	0.431	−0.031	−0.095 to 0.033	0.341
Saturated fat	−0.042	−0.228 to 0.145	0.659	−0.106	−0.401 to 0.188	0.476
Cholesterol	−0.003	−0.011 to 0.006	0.535	−0.003	−0.012 to 0.006	0.482
MUFA	0.030	−0.150 to 0.210	0.741	0.078	−0.217 to 0.373	0.604
PUFA	0.016	−0.208 to 0.239	0.891	0.024	−0.264 to 0.311	0.871
Trans fatty acids	−1.742	−5.077 to 1.592	0.303	−1.744	−5.092 to 1.604	0.305
Linoleic acid	0.006	−0.250 to 0.263	0.960	0.009	−0.336 to 0.355	0.958
Linolenic acid	1.082	−0.981 to 3.144	0.302	1.830	−0.868 to 4.529	0.182

The analytic sample: *n* = 142 participants with complete data. Model 1 adjusted for age at the preadolescent assessment, sex, village of residence, and socioeconomic status score. Model 2 additionally adjusted for total daily energy intake (kcal/day). Each dietary exposure was analyzed in a separate regression model, as continuous variables in their original units (g/day, except cholesterol, which was expressed as mg/day). CI: confidence interval; FSIQ: full-scale intelligence quotient; MUFA: monounsaturated fatty acids; PUFA: polyunsaturated fatty acids.

**Table 4:** Adjusted associations of macronutrients, fiber, and fatty acids with perceptual reasoning index (PRI).

	Model 1			Model 2		
Nutrient	B	95% CI	*p*-Value	B	95% CI	*p*-Value
Protein	−0.033	−0.100 to 0.033	0.325	−0.048	−0.144 to 0.048	0.324
Total fat	−0.008	−0.082 to 0.066	0.837	0.023	−0.127 to 0.174	0.759
Carbohydrate	−0.002	−0.030 to 0.025	0.873	0.008	−0.042 to 0.058	0.747
Dietary fiber	−0.069	−0.355 to 0.218	0.636	−0.052	−0.384 to 0.280	0.758
Total sugars	−0.013	−0.069 to 0.042	0.630	−0.010	−0.077 to 0.056	0.761
Saturated fat	−0.059	−0.252 to 0.133	0.544	−0.070	−0.374 to 0.234	0.650
Cholesterol	−0.004	−0.012 to 0.005	0.369	−0.004	−0.013 to 0.006	0.426
MUFA	0.013	−0.172 to 0.199	0.887	0.117	−0.187 to 0.421	0.447
PUFA	0.161	−0.068 to 0.391	0.167	0.312	0.021 to 0.604	0.036
Trans fatty acids	−3.044	−6.462 to 0.373	0.080	−3.029	−6.459 to 0.400	0.083
Linoleic acid	0.023	−0.242 to 0.288	0.863	0.108	−0.248 to 0.464	0.551
Linolenic acid	0.498	−1.638 to 2.634	0.645	1.340	−1.454 to 4.134	0.344

The analytic sample: *n* = 142 participants with complete data. Model 1 adjusted for age at the preadolescent assessment, sex, village of residence, and socioeconomic status score. Model 2 additionally adjusted for total daily energy intake (kcal/day). Each dietary exposure was analyzed in a separate regression model, as continuous variables in their original units (g/day, except cholesterol, which was expressed as mg/day). CI: confidence interval; PRI: perceptual reasoning index; MUFA: monounsaturated fatty acids; PUFA: polyunsaturated fatty acids.

**Table 5:** Adjusted associations of macronutrients, fiber, and fatty acids with working memory index (WMI).

	Model 1			Model 2		
Nutrient	B	95% CI	*p*-Value	B	95% CI	*p*-Value
Protein	0.050	−0.026 to 0.126	0.198	−0.027	−0.135 to 0.081	0.620
Total fat	0.061	−0.023 to 0.144	0.153	−0.096	−0.264 to 0.071	0.258
Carbohydrate	0.037	0.007 to 0.068	0.018	0.024	−0.032 to 0.079	0.397
Dietary fiber	0.510	0.195 to 0.824	0.002	0.430	0.067 to 0.794	0.021
Total sugars	0.043	−0.020 to 0.105	0.178	0.004	−0.070 to 0.079	0.907
Saturated fat	0.157	−0.061 to 0.374	0.156	−0.093	−0.432 to 0.247	0.590
Cholesterol	0.002	−0.007 to 0.012	0.666	−0.004	−0.014 to 0.007	0.491
MUFA	0.170	−0.039 to 0.380	0.110	−0.057	−0.397 to 0.283	0.741
PUFA	0.076	−0.186 to 0.339	0.567	−0.182	−0.511 to 0.148	0.278
Trans fatty acids	−2.003	−5.921 to 1.914	0.314	−2.109	−5.965 to 1.747	0.281
Linoleic acid	0.179	−0.121 to 0.479	0.239	−0.092	−0.490 to 0.306	0.649
Linolenic acid	2.637	0.247 to 5.028	0.031	1.440	−1.681 to 4.560	0.363

The analytic sample: *n* = 142 participants with complete data. Model 1 adjusted for age at the preadolescent assessment, sex, village of residence, and composite socioeconomic status score. Model 2 additionally adjusted for total daily energy intake (kcal/day). Each dietary exposure was analyzed in a separate regression model, as continuous variables in their original units (g/day, except cholesterol, which was expressed as mg/day). CI: confidence interval; MUFA: monounsaturated fatty acids; PUFA: polyunsaturated fatty acids; WMI: working memory index.

**Table 6:** Adjusted associations of macronutrients, fiber, and fatty acids with processing speed index (PSI).

	Model 1			Model 2		
Nutrient	B	95% CI	*p*-Value	B	95% CI	*p*-Value
Protein	−0.020	−0.099 to 0.059	0.613	0.018	−0.095 to 0.132	0.750
Total fat	−0.036	−0.123 to 0.051	0.415	0.013	−0.164 to 0.190	0.888
Carbohydrate	−0.017	−0.050 to 0.015	0.293	−0.011	−0.069 to 0.047	0.710
Dietary fiber	0.120	−0.217 to 0.457	0.482	0.277	−0.110 to 0.665	0.160
Total sugars	−0.054	−0.118 to 0.010	0.100	−0.051	−0.129 to 0.026	0.194
Saturated fat	−0.129	−0.355 to 0.097	0.260	−0.097	−0.454 to 0.261	0.593
Cholesterol	−0.001	−0.011 to 0.009	0.859	0.002	−0.009 to 0.013	0.748
MUFA	−0.039	−0.258 to 0.180	0.726	0.134	−0.223 to 0.491	0.460
PUFA	−0.138	−0.409 to 0.134	0.318	−0.082	−0.430 to 0.266	0.640
Trans fatty acids	−0.122	−4.195 to 3.951	0.953	−0.074	−4.148 to 4.000	0.972
Linoleic acid	−0.065	−0.377 to 0.247	0.683	0.076	−0.342 to 0.495	0.720
Linolenic acid	1.112	−1.400 to 3.624	0.383	3.325	0.081 to 6.569	0.045

The analytic sample: *n* = 142 participants with complete data. Model 1 adjusted for age at the preadolescent assessment, sex, village of residence, and composite socioeconomic status score. Model 2 additionally adjusted for total daily energy intake (kcal/day). Each dietary exposure was analyzed in a separate regression model, as continuous variables in their original units (g/day, except cholesterol, which was expressed as mg/day). CI: confidence interval; PSI: processing speed index; MUFA: monounsaturated fatty acids; PUFA: polyunsaturated fatty acids.

## Data Availability

Individual-level data from this study cannot be made publicly available due to legal restrictions.

## Supplementary Materials

The following supporting information can be downloaded at https://www.mdpi.com/article/10.3390/nu18193186/s1. Table S1: Comparison of children who completed the age 10–12 follow-up with those who did not. Table S2: Adjusted associations of macronutrients, fiber, and fatty acids with the primary cognitive outcome: Full-Scale IQ adjusted for multiple comparisons. Table S3: Adjusted associations of macronutrients, fiber, and fatty acids with the verbal comprehension index (VCI). Table S4: Sensitivity analyses of selected associations between dietary intake and cognitive outcomes.

## Abbreviations

The following abbreviations are used in this manuscript:

BMI	Body mass index
BMIZ	Body mass index z-score
CI	Confidence intervals
cm	Centimeter
FSIQ	Full-scale intelligence quotient
g	Gram
HAZ	Height-for-age z-score
IQ	Intelligence quotient
IQR	Interquartile range
kcal	Kilocalorie
kg	Kilogram
m	Meter
MUFA	Monounsaturated fatty acids
PUFA	Polyunsaturated fatty acids
PRI	Perceptual reasoning index
PSI	Processing speed index
SD	Standard deviation
SES	Socioeconomic status
VIF	Variance inflation factor
VCI	Verbal comprehension index
WMI	Working memory index
WISC-IV	Wechsler Intelligence Scale for Children-Fourth Edition
