# Supplementary material for: Associations Between Nutrient Intake and Cognitive Performance Across Multiple Domains in Healthy School-Aged Children
Source: Nutrients. 2026 Sep 26;18(19):3186. doi: 10.3390/nu18193186 (PMC13666648; doi:10.3390/nu18193186)
Supplement: Supplementary file 1 [file nutrients-18-03186-s001.zip › nutrients-4548580-supplementary.pdf]

Supplementary Table S1: Comparison of children who completed the age 10-12 follow-up with those who did not

Variable	Did not complete follow-up (n=87)	Completed follow-up (n=146)	P-value
Sex, n (%)			0.426
Male	46 (52.9)	85 (58.2)	
Female	41 (47.1)	61 (41.8)	
Village of residence, n (%)			0.028
Higher-SES village	45 (51.7)	54 (37.0)	
Lower-SES village	42 (48.3)	92 (63.0)	
Household income, n (%)			0.119
≤4000 NIS	28 (32.2)	62 (42.5)	
> 4000 NIS	59 (67.8)	84 (57.5)	
Delivery mode, n (%)			0.151
Vaginal delivery	72 (82.8)	109 (74.7)	
Cesarean section	15 (17.2)	37 (25.3)	
Father's schooling years, mean (SD)	10.5 (3.2)	10.2 (3.5)	0.453
Mother's schooling years, mean (SD)	10.7 (3.2)	10.6 (3.6)	0.790
Gestational week, mean (SD)	39.2 (1.5)	39.1 (1.39)	0.497
Birth weight (g), mean (SD)	3243.6 (445.2)	3263.6 (432.3)	0.735

Values are n (%) or mean (and standard deviation). P-values were obtained from chi-square tests for categorical variables and independent-samples t-tests for continuous variables.

NIS: New Israeli Shekel; SD: Standard Deviation; SES: socioeconomic status

Supplementary Table S2: Adjusted associations of macronutrients, fiber, and fatty acids with the primary cognitive outcome: Full-Scale IQ - adjusted for multiple comparisons

	Model 1			Model 2			
Nutrient	B	95% CI	P-value	B	95% CI	P-value	Adjusted P-value
Protein	-0.008	-0.073 to 0.057	0.815	-0.017	-0.110 to 0.077	0.727	0.949
Total fat	-0.006	-0.078 to 0.065	0.861	-0.028	-0.174 to 0.118	0.703	0.949
Carbohydrate	0.002	-0.025 to 0.029	0.872	0.006	-0.042 to 0.055	0.791	0.949
Dietary fiber	0.162	-0.114 to 0.438	0.248	0.215	-0.105 to 0.535	0.186	0.949
Total sugars	-0.021	-0.075 to 0.032	0.431	-0.031	-0.095 to 0.033	0.341	0.949
Saturated fat	-0.042	-0.228 to 0.145	0.659	-0.106	-0.401 to 0.188	0.476	0.949
Cholesterol	-0.003	-0.011 to 0.006	0.535	-0.003	-0.012 to 0.006	0.482	0.949
MUFA	0.030	-0.150 to 0.210	0.741	0.078	-0.217 to 0.373	0.604	0.949
PUFA	0.016	-0.208 to 0.239	0.891	0.024	-0.264 to 0.311	0.871	0.950
Trans fatty acids	-1.742	-5.077 to 1.592	0.303	-1.744	-5.092 to 1.604	0.305	0.949
Linoleic acid	0.006	-0.250 to 0.263	0.960	0.009	-0.336 to 0.355	0.958	0.958
Linolenic acid	1.082	-0.981 to 3.144	0.302	1.830	-0.868 to 4.529	0.182	0.949

The analytic sample: n=142 participants with complete data.

Model 1 was adjusted for age at the preadolescent assessment, sex, village of residence, and composite socioeconomic status (SES) score. Model 2 additionally adjusted for total daily energy intake (kcal/day). Each dietary exposure was analyzed in a separate regression model. Nutrient intakes were modeled as continuous variables in their original units (g/day, except cholesterol, which was expressed as mg/day). CI, confidence interval; IQ, intelligence quotient; MUFA, monounsaturated fatty acids; PUFA, polyunsaturated fatty acids.

Adjusted P values represent Benjamini–Hochberg false discovery rate-adjusted P values across the 12 prespecified Model 2 nutrient–FSIQ comparisons.

Supplementary Table S3: Adjusted associations of macronutrients, fiber, and fatty acids with the verbal comprehension index (VCI)

	Model 1			Model 2		
Nutrient	B	95% CI	P-value	B	95% CI	P-value
Protein	0.018	-0.041 to .077	0.543	0.009	-0.076 to .094	0.835
Total fat	0.007	-0.058 to .072	0.826	-0.047	-0.179 to .086	0.487
Carbohydrate	0.008	-0.016 to .032	0.519	0.004	-0.040 to .048	0.846
Dietary fiber	0.088	-0.164 to .341	0.491	0.062	-0.231 to .355	0.674
Total sugars	-0.007	-0.056 to .042	0.779	-0.023	-0.081 to .036	0.445
Saturated fat	0.033	-0.137 to .203	0.701	-0.025	-0.293 to .244	0.856
Cholesterol	-0.002	-0.010 to .005	0.556	-0.004	-0.012 to .004	0.321
MUFA	0.032	-0.132 to .196	0.699	-0.028	-0.296 to .241	0.839
PUFA	0.028	-0.176 to .232	0.785	-0.022	-0.283 to .239	0.869
Trans fatty acids	-1.469	-4.510 to 1.572	0.341	-1.493	-4.541 to 1.555	0.334
Linoleic acid	-0.002	-0.236 to .232	0.986	-0.095	-0.409 to 0.218	0.549
Linolenic acid	1.153	-0.724 to 3.030	0.226	1.291	-1.171 to 3.754	0.302

The analytic sample: n=142 participants with complete data.

Model 1 was adjusted for age at the preadolescent assessment, sex, village of residence, and composite socioeconomic status score. Model 2 additionally adjusted for total daily energy intake (kcal/day). Each dietary exposure was analyzed in a separate regression model. Nutrient intakes were modeled as continuous variables in their original units (g/day, except cholesterol, which was expressed as mg/day). CI: confidence interval; VCI: Verbal Comprehension Index; MUFA, monounsaturated fatty acids; PUFA, polyunsaturated fatty acids.

Supplementary Table S4: Sensitivity analyses of selected associations between dietary intake and cognitive outcomes

Association	Analysis/exposure specification	B	95% CI	P-value
Dietary fiber-WMI	Primary energy-adjusted model	0.430	0.067 to 0.794	0.021
	Log-transformed dietary fiber	7.412	1.455 to 13.368	0.015
	Excluding influential observations	0.362	0.005 to 0.720	0.047
Linolenic acid-PSI	Primary energy-adjusted model	3.325	0.081 to 6.569	0.045
	Excluding PSI outlier	3.783	0.656 to 6.910	0.018
	Log-transformed linolenic acid	4.416	-0.678 to 9.510	0.089
	Linolenic acid as % of total energy	5.453	-1.279 to 12.184	0.112
PUFA-PRI	Primary energy-adjusted model	0.312	0.021 to 0.604	0.036
	PUFA as % of total energy	0.612	-0.092 to 1.316	0.088
	Log-transformed PUFA	1.384	-1.681 to 4.449	0.373
Trans fatty acids- PRI	Continuous, energy-adjusted model	-3.029	-6.459 to 0.400	0.083
	Any vs no intake, energy-adjusted model	-6.290	-10.766 to -1.814	0.006

B: unstandardized regression coefficient; CI: confidence interval; PUFA, polyunsaturated fatty acids; PRI, Perceptual Reasoning Index; PSI, Processing Speed Index; WMI, Working Memory Index. Primary energy-adjusted models (Model 2) were adjusted for age at the preadolescent assessment, sex, village of residence, composite socioeconomic status, and total daily energy intake. Each dietary exposure was analyzed in a separate regression model. Nutrient intakes were modeled as continuous variables in their original units unless otherwise indicated. For transformed or alternative exposure specifications, regression coefficients are interpreted on the corresponding exposure scale and are therefore not directly comparable across rows. Sensitivity analyses were exploratory and were conducted for selected associations identified in the secondary cognitive-domain analyses.
